# Targeting lncRNA PSMA3-AS1, a Prognostic Marker, Suppresses Malignant Progression of Oral Squamous Cell Carcinoma

**DOI:** 10.1155/2021/3138046

**Published:** 2021-08-19

**Authors:** Xinghua Cao, Kefeng Luan, Jie Yang, Yundong Huang

**Affiliations:** ^1^Department of Stomatology, Affiliated Hospital of Weifang Medical University, Weifang, 261031 Shandong, China; ^2^Department of Stomatology, Weifang Second People's Hospital, Weifang, 261041 Shandong, China

## Abstract

**Objective:**

Oral squamous cell carcinoma (OSCC) represents the most common maxillofacial malignancy. This study elucidated the clinicopathological value and molecular mechanisms of PSMA3 antisense RNA 1 (PSMA3-AS1) in OSCC.

**Methods:**

Totally, 135 OSCC patients were recruited. PSMA3-AS1 expression and its prognostic value were assessed in this cohort. si-PSMA3-AS1 was transfected into HN4 and CAL-27 OSCC cells. Then, cell proliferation was evaluated by CCK-8, colony formation, and EdU staining. Migration and invasion were investigated through wound healing, transwell, and western blot. The PSMA3-AS1/miR-136-5p and miR-136-5p/FN1 interactions were validated by dual luciferase report, real-time quantitative polymerase chain reaction (RT-qPCR), and western blot.

**Results:**

PSMA3-AS1 upregulation was determined in OSCC tissues. The upregulation indicated pessimistic patients' outcomes. Multivariate Cox regression analyses confirmed PSMA3-AS1 as an independent prognostic indicator. Its upregulation was also found in OSCC cells. Under transfection with si-PSMA3-AS1, proliferation, migration, and invasion were all restrained in HN4 and CAL-27 OSCC cells. Furthermore, its knockdown induced the increase in E-cadherin expression and the reduction in N-cadherin and Vimentin expression. PSMA3-AS1 was a sponge of miR-136-5p. Mutual inhibition was found between two and the interactions were confirmed by dual luciferase report. It was confirmed that FN1 was a target of miR-136-5p. FN1 expression was increased by miR-136-5p inhibitors, which was lessened by si-PSMA3-AS1 cotransfection.

**Conclusion:**

Collectively, PSMA3-AS1 as a risk factor facilitated malignant behaviors of OSCC cells, related to the miR-136-5p/FN1 axis. Hence, PSMA3-AS1 as a potential therapeutic target for OSCC deserved further exploration.

## 1. Introduction

Oral squamous cell carcinoma (OSCC) represents the most common maxillofacial malignancy worldwide, accounting for approximately 90% among head and neck malignancies [1]. More than 300,000 new cases occur each year globally [2]. Meanwhile, death cases exceed 140,000 each year. The five-year survival rate is <50% [3]. The main therapeutic choices contain surgery, radiotherapy, and chemotherapy [4]. However, OSCC exhibits high invasiveness, low response to antitumor therapies, early recurrence, and undesirable clinical outcomes [5]. Putting together, it requires discovering innovative molecular targets and developing new therapeutic markers that possess higher potency.

lncRNA is a noncoding RNA with >200 nucleotides in length [6]. Studies have shown that lncRNA participates in mediating cancer-related miscellaneous physiological as well as pathological processes [7]. Data show that during tumorigenesis process, lncRNA as a competitive RNA (ceRNA) competitively binds to miRNA, thereby regulating and interacting with each other as well as affecting downstream mRNA expression [8]. As reported in previous research, PSMA3-AS1 functions as an oncogenic lncRNA [9]. PSMA3-AS1 upregulation may facilitate the progress of multiple myeloma [9], esophageal cancer [10], lung cancer [11], colorectal cancer [12], and glioma [13]. Mechanically, PSMA3-AS1 accelerates lung cancer growth and invasion through sponging miR-4504 [11]. PSMA3-AS1 as a sponge of miR-4429 induces colorectal cancer cell migration and invasion [12]. PSMA3-AS1 facilitates malignant phenotypes of esophageal cancer via the miR-101/EZH2 axis [10]. Nevertheless, it remains undiscovered concerning the roles and mechanisms of PSMA3-AS1 during OSCC. Here, we aimed to observe the roles of PSMA3-AS1 on OSCC progress and assess the potential of PSMA3-AS1 expression as an alternative marker for accurately predicting outcomes and as an underlying target for OSCC therapy. Moreover, this study observed the underlying mechanisms of PSMA3-AS1 in OSCC cells.

## 2. Materials and Methods

### 2.1. Patients and Specimens

Totally, 135 patients who underwent radical resection of OSCC in Affiliated Hospital of Weifang Medical University from January 2013 to August 2014 were selected as the research objects. The inclusion criteria were as follows: (1) pathological examination that confirmed the diagnosis; (2) first diagnosis; (3) no radiotherapy, chemotherapy, or other treatment; and (4) complete clinical and follow-up information. The normal oral mucosa tissue over 2 cm from the edge of the tumor was selected as the control. Fresh tissues that were frozen at −80°C after the operation were used for further analyses. The study was approved by the Ethics Committee of the Affiliated Hospital of Weifang Medical University (2013005), and an informed consent form was signed with the patient or family member.

### 2.2. Real-Time Quantitative Polymerase Chain Reaction (RT-qPCR)

The total RNA of tissue or cell specimens was extracted, and the extraction method was carried out according to the Trizol operating instructions (Beyotime, Shanghai, China). The reverse transcription reaction system contained 1 *μ*L RT Enzyme Mix I, 1 *μ*L Random 6 mers, 4 *μ*L 5x PrimeScript Buffer, 1 *μ*L Oligo dT Primer, and RNase-free H_2_O. cDNA was collected and PCR reaction was performed with SYBR.  Table 1 lists the primers for PSMA3-AS1, miR-136-5p, GAPDH, and U6. The PCR reaction program included predenaturation at 95°C for 30 s, denaturation at 95°C for 5 s, and annealing extension at 60°C for 60 s, totally 40 cycles. The result was calculated on the basis of the 2^-*ΔΔ*Ct^ method.

### 2.3. Cell Culture

Normal human oral mucosal epithelial cells (NHOK) and four OSCC cells HN4, HN6, SCC-25, and CAL-27 were purchased from Shanghai Huiying Biological Technology Co., Ltd. (China). They were cultured with DMEM medium (Gibco, USA) containing 10% fetal bovine serum (Gibco, USA) in an incubator at 37°C and 5% CO_2_ saturated humidity.

### 2.4. Transfection

Small interfering RNAs (siRNAs) against PSMA3-AS1 (si-PSMA3-AS1) and their negative control (si-NC), miR-136-5p mimics, miR-136-5p inhibitors, and miRNA negative control (miR-NC) were all constructed and synthesized by Shanghai GenePharma Technology Co., Ltd. (China). HN4 and CAL-27 cells in the logarithmic growth phase were selected and seeded in a 6 cm Petri dish. After 24 h of culture, si-PSMA3-AS1, si-NC, miR-136-5p mimics, miR-136-5p inhibitors, and miR-NC were, respectively, transfected into HN4 and CAL-27 cells through Lipofectamine 2000 (Invitrogen, USA) referring to the Lipofectamine 2000 kit instructions. After 24 h of transfection, follow-up assays were presented.

### 2.5. Cell Counting Kit-8 (CCK-8)

si-NC- and si-PSMA3-AS1-transfected cells were seeded in 96-well plates (2 × 10^3^ cells/well). Each group had 4 multiple holes. After continuing the culture for 0, 24, 48, 72, and 96 h, the CCK-8 kit (Dojindo, Japan) was added for detection of cell viability. The specific operation was carried out following the kit instructions. Utilizing microplate reader, we detected the absorbance value at 450 nm wavelength.

### 2.6. Colony Formation Assay

HN4 and CAL-27 cells were digested with 0.25% trypsin into a single cell suspension. 500 cells transfected with si-NC or si-PSMA3-AS1 were seeded in a 60 mm cell culture dish. After 3 days of continuous cultivation, the culture solution was changed. 3 mL culture medium was added to each dish and continued to culture for 8 days. Then, the culture was terminated. Cells were stained by Giemsa after the cells were fixed. Pictures were taken and the cell colonies were counted.

### 2.7. 5-Ethynyl-2′-deoxyuridine (EdU) Staining

HN4 and CAL-27 cells transfected with si-NC and si-PSMA3-AS1 were seeded on 96-well plates (8 × 10^3^ cells/well) and cultured overnight. EdU solution (Sigma, USA) was diluted with complete medium at a ratio of 1000 : 1 to prepare an appropriate amount of 50 *μ*mol/L EdU medium. 100 *μ*L 50 *μ*mol/L EdU medium was added to each well for 2 h incubation. The culture medium was discarded, and the cells were washed with PBS buffer twice for 5 min each time. 100 *μ*L cell fixative was added to each well and incubated for 30 min at 37°C. Following adding 2 mg/mL glycine, cells were incubated for 5 min on a decolorizing shaker. 100 *μ*L PBS buffer was added to each well and continued to incubate for 5 min on a decolorizing shaker. After adding 100 *μ*L 1x Apollo staining reaction solution to each well, cells were incubated for 30 min at 37°C in the dark. The staining reaction solution was removed, and 100 *μ*L penetrant was added. Samples were washed twice on a decolorizing shaker, each for 10 min. After exhausting the culture medium, the cells were fixed with 0.5 mL fixative for 10 min. The fixative was removed and cells were washed twice with PBS, 3 min each time. Cells were then stained with 0.5 mL Hoechst staining solution (Abcam, USA) for 5 min. After being washing twice with PBS, 3 min each time, antifluorescence quenching mounting solution was dropwise added to cover the cover glass. Images were observed under a fluorescence microscope (Olympus, Japan).

### 2.8. Wound Healing Assay

This study collected HN4 and CAL-27 cells that were transfected with si-NC or si-PSMA3-AS1 for 48 h. Cells were resuspended in serum-free medium. The cell suspension was seeded on a 6-well plate. After 24 h of inoculation, a 200 *μ*L pipette tip was used for scratching the 6-well plate. After washing 3 times with PBS, the migration of cells was observed and taken pictures at 0 h and 48 h after the scratch. The migratory rate = (0 h width − 48 h width)/0 h width × 100%.

### 2.9. Transwell Assay

Transwell assays were used for measuring migration as well as invasion. Matrigel (Corning, USA) was used for coating before the invasion experiment, and the coating step was omitted before the migration experiment. The rest of the steps was the same and was briefly described as follows: the cells in si-NC and si-PSMA3-AS1 groups were suspended in a serum-free cell culture medium. Then, 200 *μ*L cells were drawn and planted in the upper chamber. At the same time, 500 *μ*L serum-containing cell culture medium was added to the lower chamber. The chamber was placed in a CO_2_ incubator at 37°C for 24 h. The cells were fixed with methanol and stained with crystal violet. Five fields were randomly selected under the microscope (Olympus, Japan) to count the number of cells penetrating the membrane.

### 2.10. Dual Luciferase Report

The wild-type (WT) or mutant (MUT) gene sequences were cloned into the pmirGLO plasmid to obtain pmirGLO-PSMA3-AS1 WT/MUT and pmirGLO-FN1 WT/MUT. Using Lipofectamine™ 2000 transfection reagent, the recombinant plasmids combining with miR-NC or miR-136-5p mimics were transferred into HN4 or CAL-27 cells. After 48 h, the luciferase activity was detected according to the steps described in the dual luciferase reporter kit (Beyotime, Shanghai, China).

### 2.11. Western Blot

Western blot method was used for determining E-cadherin, N-cadherin, Vimentin, and FN1 proteins. The steps were briefly described as follows: the total protein was extracted from cells in each group. The amount of protein loaded in each well was 40 *μ*g. The electrophoresis voltage was 90 V, and the electrophoresis at constant voltage was 0.5 h. Under electrophoresis at 120 V, constant voltage lasted for 2 h. The NC membrane was cut to the same size as the separation glue. The membrane was transferred under the condition of 60 V constant pressure; the transfer device was performed at 4°C. And the transfer time was set to 60 min. The membrane was closed by 5% skimmed milk powder. The membrane was incubated with primary antibodies against E-cadherin (1 : 1000; #3195; CST, USA), N-cadherin (1 : 1000; #13116; CST), Vimentin (1 : 1000; #5741; CST), FN1 (1 : 1000; #26836; CST), and GAPDH (1 : 1000; #5174; CST) overnight and incubated with secondary antibodies (#7075; CST) for 2 h. Color was developed with electrochemical luminescence method (Millipore, USA). As a result, ImageJ was used for analyzing the OD value of the band with GAPDH as an internal reference.

### 2.12. Statistical Analysis

GraphPad Prism 5, SPSS 23.0, and R software were utilized for statistical analysis. Quantitative data were expressed by mean ± standard deviation. Comparisons between groups were assessed via Student's *t*-test or one-way analysis of variance. Correlation between PSMA3-AS1 expression and clinicopathological parameters was analyzed through the chi-square test. Kaplan-Meier overall survival (OS) and disease-free survival (DFS) curves were depicted between high and low PSMA3-AS1 expression subgroups. The differences in survival between subgroups were confirmed through log-rank test. Multivariate Cox regression analyses were used for assessing which parameters independently predicted subjects' survival. *P* value < 0.05 was considered statistically significant.

## 3. Results

### 3.1. lncRNA PSMA3-AS1 Upregulation as a Prognostic Factor of OSCC

**Figure 1:**
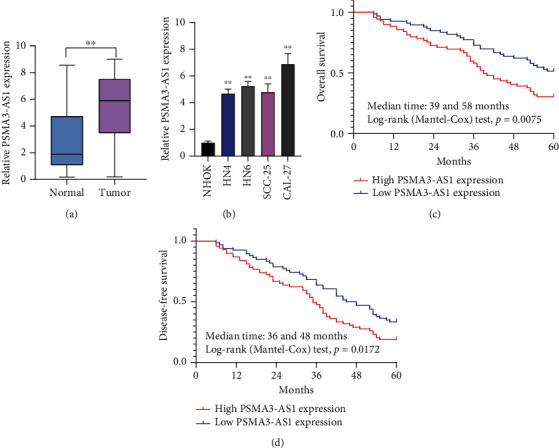
Expression and prognostic value of PSMA3-AS1 expression in OSCC. (a) RT-qPCR for PSMA3-AS1 upregulation in OSCC than normal tissue specimens. Compared to normal tissues, ^∗∗^*P* < 0.01. (b) RT-qPCR results of PSMA3-AS1 overexpression in HN4, HN6, SCC-25, and CAL-27 OSCC cell lines than NHOK normal oral mucosal epithelial cells. Compared to NHOK cells, ^∗∗^*P* < 0.01. (c) OS analyses between high and low PSMA3-AS1 expression OSCC subgroups. (d) DFS analyses between high and low PSMA3-AS1 expression OSCC subgroups. The differences in survival between subgroups were compared by log-rank tests.

Here, 135 OSCC subjects were recruited. PSMA3-AS1 overexpression was displayed in OSCC than normal tissue specimens (Figure 1(a)), indicating that this lncRNA might be in relation to OSCC progress. We compared PSMA3-AS1 expression in NHOK normal oral mucosal epithelial cells with four OSCC cell lines HN4, HN6, SCC-25, and CAL-27 (Figure 1(b)). Data were indicative of the upregulation of PSMA3-AS1 in OSCC than normal cell lines. Then, the prognostic value of PSMA3-AS1 upon OSCC was under evaluation.  Table 2 lists that PSMA3-AS1 expression did not exhibit distinct associations with gender, age, and tumor size among OSCC subjects. Nevertheless, PSMA3-AS1 expression was in relation to distant metastasis as well as TNM stage, which was indicative that this lncRNA might participate in malignant progress of OSCC. We assessed whether PSMA3-AS1 expression was predictive of subjects' outcomes. In Figure 1(c), PSMA3-AS1 upregulation was indicative of shorter OS time in comparison to its downregulation. In the meanwhile, subjects with its low expression experienced pessimistic DFS time (Figure 1(d)). To verify whether this lncRNA was independently predictive of OSCC outcomes, multivariate Cox regression analyses were carried out. Following incorporating gender, age, tumor size, distant metastasis, TNM stage, and PSMA3-AS1 expression, our data confirmed that TNM stage and PSMA3-AS1 expression were both independently predictive of subjects' outcomes (Table 3). Collectively, PSMA3-AS1 upregulation was a prognostic indicator of OSCC.

### 3.2. PSMA3-AS1 Knockdown Weakens OSCC Cellular Proliferative Potential

**Figure 2:**
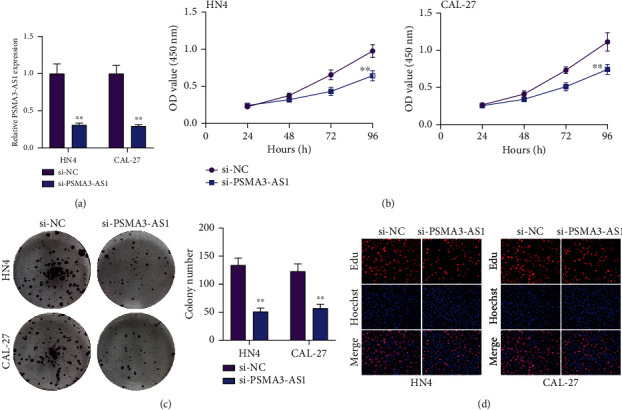
Assessment of OSCC cellular proliferative potential following PSMA3-AS1 knockdown. (a) Examination of PSMA3-AS1 expression in HN4 as well as CAL-27 cells under transfection with si-PSMA3-AS1 or si-NC. Compared to si-NC, ^∗∗^*P* < 0.01. (b) CCK-8 assay for cell viability of HN4 and CAL-27 cells at 0, 24, 48, 72, and 96 h following transfection with si-PSMA3-AS1 or si-NC. Compared to 24 h, ^∗∗^*P* < 0.01. (c) The colony formation number of HN4 and CAL-27 cells transfected with si-PSMA3-AS1 or si-NC. Compared to si-NC, ^∗∗^*P* < 0.01. (d) EdU staining for HN4 and CAL-27 cells under transfection with si-PSMA3-AS1 or si-NC.

This study explored the molecular basis concerning high PSMA3-AS1 expression for promotion of OSCC subjects' poor outcomes. To silence PSMA3-AS1, siRNAs against PSMA3-AS1 were transfected into HN4 and CAL-27 OSCC cells. The transfection results were examined via RT-qPCR. In Figure 2(a), PSMA3-AS1 displayed lowered expression in si-PSMA3-AS1-transfected HN4 and CAL-27 OSCC cells, indicating that this lncRNA was successfully silenced. Whether PSMA3-AS1 affected OSCC cellular proliferation was under assessment. CCK-8 assay was utilized for cell viability at 0, 24, 48, 72, and 96 h after transfection with si-PSMA3-AS1 or si-NC. In Figure 2(b), silencing PSMA3-AS1 exhibited the restrained functions on HN4 cellular viability. The similar data were observed in CAL-27 OSCC cells. Clone formation capacities of OSCC cells were then assessed. In comparison to si-NC transfection, si-PSMA3-AS1 transfection displayed the decreased colony number of HN4 cells as well as CAL-27 cells (Figure 2(c)). EdU staining was used for assessing cellular proliferation. As depicted in Figure 2(d), proliferative abilities were markedly lessened by PSMA3-AS1 knockdown. Hence, PSMA3-AS1 exerted a stimulative role on OSCC proliferation.

### 3.3. Silencing PSMA3-AS1 Lessens OSCC Cellular Migratory and Invasive Abilities

**Figure 3:**
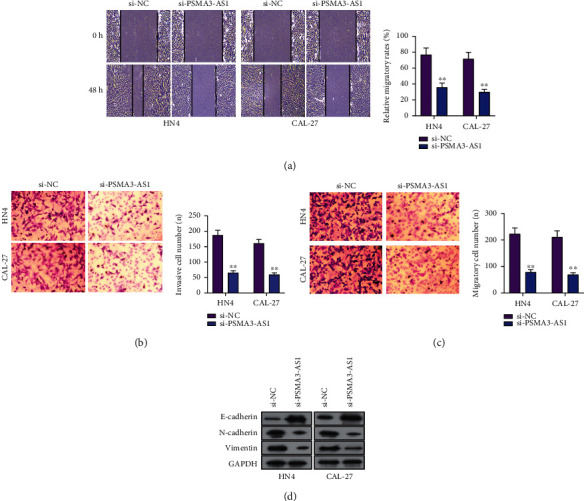
The restraining functions of PSMA3-AS1 knockdown on OSCC cellular migratory and invasive capacities. (a) Measurement of wound distance in HN4 and CAL-27 cells with si-PSMA3-AS1 or si-NC transfection at 0 and 48 h. (b) Calculation of invasive HN4 and CAL-27 cell number under transfection with si-PSMA3-AS1 or si-NC. (c) Evaluation of migratory HN4 and CAL-27 cell number following being transfected with si-PSMA3-AS1 or si-NC. (d) Western blot for E-cadherin, N-cadherin, and Vimentin proteins in HN4 and CAL-27 cells with si-PSMA3-AS1 or si-NC transfection. In comparison to si-NC, ^∗∗^*P* < 0.01.

Migration and invasion are key manifestations of the malignant behaviors of OSCC. This study evaluated whether PSMA3-AS1 influenced migratory as well as invasive abilities in OSCC cells. According to wound healing assay data, the scratch of si-PSMA3-AS1-transfected HN4 and CAL-27 cells showed wider distance than si-NC transfection (Figure 3(a)). The data were indicative of the inhibitory roles of PSMA3-AS1 knockdown on migratory ability of OSCC cells. Transwell assays were carried out for evaluation of migration in OSCC cells. In Figure 3(b), compared to the si-NC group, the si-PSMA3-AS1 group exhibited the lowered number of invasive HN4 and CAL-27 cells. Also, the migratory OSCC cell number had a distinct reduction by PSMA3-AS1 knockdown (Figure 3(c)). Epithelial-to-mesenchymal transition (EMT) process exerts a key function in cancer migration as well as metastases. Here, E-cadherin, N-cadherin, and Vimentin proteins were detected in HN4 and CAL-27 cells through western blot. Consequently, si-PSMA3-AS1 transfection markedly augmented E-cadherin expression as well as lessened N-cadherin and Vimentin expression in OSCC cells (Figure 3(d)). This indicated that EMT process of OSCC cells was obstructed by silencing PSMA3-AS1. Taken together, PSMA3-AS1 knockdown weakened migratory as well as invasive capacities of OSCC cells.

### 3.4. PSMA3-AS1 Acts as a Sponge of miR-136-5p That Is Downregulated in OSCC

**Figure 4:**
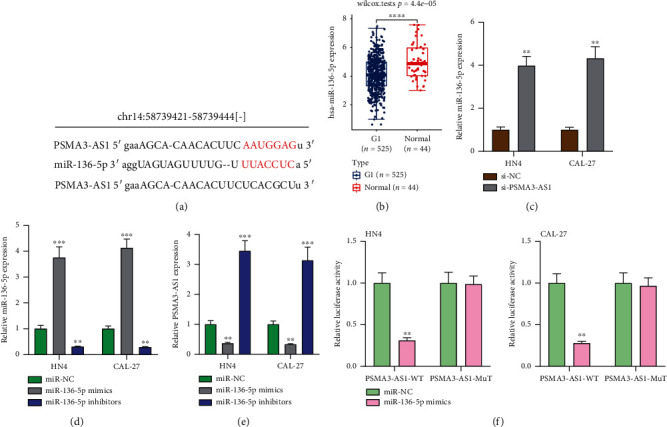
PSMA3-AS1 acts as a sponge of miR-136-5p that is downregulated in OSCC. (a) Schematic diagram of the binding sites of PSMA3-AS1 and miR-136-5p. (b) Lowered miR-136-5p expression in OSCC than normal tissues from TCGA database. Compared to normal tissues, ^∗∗∗∗^*P* < 0.0001. (c) Higher miR-136-5p expression in HN4 and CAL-27 cells transfected with si-PSMA3-AS1 than si-NC. Compared to si-NC, ^∗∗^*P* < 0.01. (d) Verification of miR-136-5p expression in HN4 and CAL-27 cells with miR-136-5p mimic or inhibitor transfection. Compared to miR-NC, ^∗∗^*P* < 0.01 and ^∗∗∗^*P* < 0.001. (e) Assessment of PSMA3-AS1 expression in HN4 and CAL-27 cells transfected with miR-136-5p mimics or inhibitors. Compared to miR-NC, ^∗∗^*P* < 0.01 and ^∗∗∗^*P* < 0.001. (f) Relative luciferase activity in PSMA3-AS1-WT or PSMA3-AS1-Mut HN4 and CAL-27 cells transfected with miR-136-5p mimics and miR-NC. Compared to miR-NC, ^∗∗^*P* < 0.01.

Under confirmation, lncRNA acts as a sponge of miRNA thereby being involved in cancer progress. It was predicted that there were binding sites between PSMA3-AS1 and miR-136-5p via the RNAhybrid 2.12 (http://www.targetscan.org/worm_52/) (Figure 4(a)) [14]. Contrary to PSMA3-AS1 expression, miR-136-5p displayed downregulation in OSCC than in normal tissues in TCGA database (Figure 4(b)). Then, we validated the interactions between PSMA3-AS1 and miR-136-5p in OSCC cells. In Figure 4(c), miR-136-5p showed an increased expression in HN4 and CAL-27 cells with PSMA3-AS1 knockdown, indicating that PSMA3-AS1 upregulation might restrain miR-136-5p expression. Oppositely, we investigated whether miR-136-5p affected PSMA3-AS1 expression in OSCC cells. After miR-136-5p mimics as well as inhibitors were separately transfected into HN4 and CAL-27 cells, miR-136-5p expression was firstly assessed. In Figure 4(d), compared to miR-NC transfection, higher miR-136-5p expression was confirmed in miR-136-5p mimic-treated OSCC cells. Meanwhile, miR-136-5p expression showed distinctly lowered expression under transfection with miR-136-5p inhibitors. Afterwards, we determined PSMA3-AS1 expression in OSCC cells. There was lowered PSMA3-AS1 expression in HN4 and CAL-27 cells treated with miR-136-5p mimics than miR-NC (Figure 4(e)). Oppositely, elevated expression of PSMA3-AS1 was verified following treatment with miR-136-5p inhibitors. Thus, there was mutual inhibition between PSMA3-AS1 and miR-136-5p in OSCC. To confirm whether PSMA3-AS1 directly bound to the binding sites of miR-136-5p, dual luciferase report was carried out. In the PSMA3-AS1-WT HN4 cells, lowered luciferase activity was found under miR-136-5p mimic transfection than miR-NC transfection; however, there was no significant difference in luciferase activity between miR-136-5p mimic and miR-NC transfection in PSMA3-AS1-Mut HN4 cells (Figure 4(f)). Similar findings were verified in CAL-27 cells. Collectively, PSMA3-AS1 acted as a sponge of miR-136-5p in OSCC.

### 3.5. PSMA3-AS1 Knockdown Indirectly Lowers FN1 Expression via Upregulating miR-136-5p in OSCC

**Figure 5:**
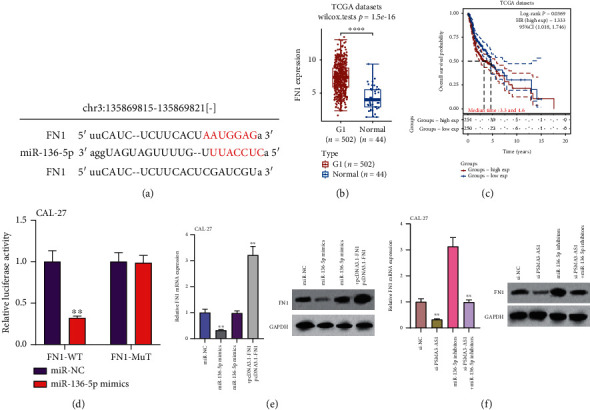
PSMA3-AS1 knockdown indirectly lowers FN1 expression via upregulating miR-136-5p in OSCC. (a) Schematic diagram of miR-136-5p and FN1 binding sites. (b) FN1 upregulation in OSCC than normal tissue specimens from TCGA database. Compared to normal tissues, ^∗∗∗∗^*P* < 0.0001. (c) Overall survival analyses between FN1 high and low expression subgroups. The differences between subgroups were tested by log-rank tests. (d) Relative luciferase activity in CAL-27 cells with FN1-WT or FN1-Mut following transfection with miR-NC or miR-136-5p mimics. Compared to miR-NC, ^∗∗^*P* < 0.01. (e) FN1 mRNA and protein expressions in CAL-27 cells transfected with miR-136-5p mimics and/or pcDNA3.1-FN1. (f) FN1 mRNA and protein expression in CAL-27 cells under transfection with si-PSMA3-AS1 and/or miR-136-5p inhibitors. Compared to si-NC or miR-136-5p inhibitors, ^∗∗^*P* < 0.01.

The targeted mRNAs of miR-136-5p were predicted by the RNAhybrid 2.12. Among them, FN1 mRNA was an underlying target of miR-136-5p (Figure 5(a)). FN1 mRNA was validated in TCGA database. Opposite to miR-136-5p expression, FN1 displayed elevated expression in OSCC than normal tissue specimens (Figure 5(b)). The prognostic value of FN1 mRNA was then evaluated.  Figure 5(c) demonstrated that FN1 might serve as an oncogene for OSCC. Subjects with high FN1 expression were indicative of pessimistic outcomes. Dual luciferase report was presented to confirm the interactions of miR-136-5p and FN1. In CAL-27 cells with PSMA3-AS1-WT, miR-136-5p mimics distinctly reduced relative luciferase activity (Figure 5(d)). However, for cells with PSMA3-AS1-Mut, miR-136-5p mimics did not change relative luciferase activity. The data confirmed the direct interactions between miR-136-5p and FN1. Afterwards, we assessed whether PSMA3-AS1 affected FN1 expression by miR-136-5p in OSCC. In Figure 5(e), FN1 mRNA expression was reduced by miR-136-5p mimics in CAL-27 cells. The reduction was ameliorated by pcDNA3.1-FN1 cotransfection. Meanwhile, we examined FN1 protein expression. Consistently, FN1 protein showed a decreased expression in CAL-27 cells with miR-136-5p mimics. This decrease was improved under cotransfection of pcDNA3.1-FN1. Following transfection with si-PSMA3-AS1, lowered FN1 expression was confirmed in CAL-27 cells at the mRNA and protein levels (Figure 5(f)). Nevertheless, si-PSMA3-AS1 lessened the stimulative roles of miR-136-5p inhibitors on FN1 expression. Collectively, PSMA3-AS1 knockdown indirectly lowered FN1 expression via upregulating miR-136-5p in OSCC.

## 4. Discussion

In this study, we proposed a novel lncRNA PSMA3-AS1 that exhibited upregulation in OSCC. It acted as an independent risk factor for this malignancy. Moreover, it was involved in OSCC malignant biological properties containing proliferation, migration, and invasion, which was in relation with the miR-136-5p/FN1 axis.

OSCC subjects' treatment is mainly on the basis of clinicopathological indicators like TNM and grade [15]. Nevertheless, it has been under confirmation that novel molecular biomarkers may prolong survival time. Thus, it requires probing novel molecules that may characterize subjects' risk stratifications. Here, PSMA3-AS1 showed upregulation in OSCC than normal specimens. Its expression could be utilized for independently predicting patients' outcomes. Previously, Xu et al. reported that circulating PSMA3-AS1 exhibited distinct correlations to multiple myeloma subjects' prognosis [9]. Qiu et al. found that elevated PSMA3-AS1 expression was in relation with tumor size, distant metastases, and pessimistic outcomes for esophageal cancer [10]. Li et al. demonstrated that its expression showed positive correlations to stage, metastasis, and poor outcomes for lung cancer [11]. As reported in the study of Wang et al., PSMA3-AS1 expression was in correlation to stage, lymph node metastases, and OS in non-small-cell lung carcinoma [16]. PSMA3-AS1 was a prognostic marker for diffuse large B-cell lymphoma [17]. Thus, PSMA3-AS1 may be an oncogene for OSCC.

Our data confirmed that targeting PSMA3-AS1 weakened OSCC cell proliferative characteristics. Lymph node metastasis is a critical clinical feature of OSCC, as the primary cause of postoperative recurrence in OSCC patients [18]. Tumor cell metastasis contains the corresponding sequence and related steps. Many of these steps are affected by EMT. The ability of cells to obtain mesenchymal properties enhances cell migration and invasion, induces the stem cell characteristics of tumor cells, and inhibits cell apoptosis and programmed senescence, allowing cells to metastasize [19–21]. EMT is a key for therapy resistance, recurrence, and distant metastases [22]. The main characteristics of EMT in tissues or organs include the decrease in E-cadherin expression and the increase in Vimentin and N-cadherin expression [23]. Here, targeting PSMA3-AS1 restrained migratory and invasive features of OSCC cells, which elevated E-cadherin level as well as lessened Vimentin and N-cadherin levels in OSCC, thereby inactivating EMT process.

PSMA3-AS1 acted as a sponge of miR-136-5p. There were mutual inhibitory interactions between PSMA3-AS1 and miR-136-5p. This miRNA downregulation was determined in OSCC, confirmed by previous research [18]. Its downregulation has been found in various malignancies like thyroid carcinoma [24]. Overexpressing miR-136-5p lessened malignant progress of thyroid carcinoma [24]. Moreover, Li et al. found that forced miR-136-5p suppressed proliferation as well as metastases of renal cell carcinoma [25]. Its anticancer effect was also observed in triple-negative breast cancer [26]. Geng et al. reported that hsa_circ_0014130 facilitated non-small-cell lung cancer progress by acting as a sponge of miR-136-5p [27]. Furthermore, hypomethylated PlncRNA-1 enhanced bladder carcinoma development via sponging miR-136-5p [28]. Thus, miR-136-5p may exert an anticancer function. Nevertheless, its functions in OSCC required in-depth exploration. FN1 was a target of miR-136-5p. Its expression was elevated in OSCC, consistent with previous research [29]. FN1 may act as a risk factor of OSCC [30, 31]. After investigation, PSMA3-AS1 mediated the miR-136-5p/FN1 axis, thereby accelerating OSCC progress.

Several limitations should be pointed out. First, the prognostic value of PSMA3-AS1 expression should be verified in a prospective cohort. Second, it requires investigating the carcinogenic role of PSMA3-AS1 in vivo. Third, more experiments will be presented for confirming the interactions of PSMA3-AS1/miR-136-5p/FN1.

## 5. Conclusion

Collectively, in the OSCC cohort, PSMA3-AS1 was independently predictive of patients' outcomes. PSMA3-AS1 upregulation was implicated in carcinogenesis as well as cancer cellular proliferative, migratory, and invasive features. In terms of mechanisms, PSMA3-AS1 could play a carcinogenic effect through the miR-136-5p/FN1 axis. Hence, targeting carcinogenic PSMA3-AS1 offered novel therapeutic thought against OSCC.

## Figures and Tables

**Table 1:** The primers used in this study for RT-qPCR.

Names	Sequences (5′-3′)
PSMA3-AS1: F	TTCCTCCAGGACAGCACCTAGT
PSMA3-AS1: R	CGTCTCTGATGTGGCTTATACGA
miR-136-5p: F	ACACTCCAGCTGGGACTCCATTTGTTTT
miR-136-5p: R	CCAGTGCAGGGTCCGAGGT
FN1: F	CGGTGGCTGTCAGTCAAAG
FN1: R	AAACCTCGGCTTCCTCCATAA
GAPDH: F	CGGAGTCAACGGATTTGGTCGTAT
GAPDH: R	AGCCTTCTCCATGGTGGTGAAGAC
U6: F	ATTGGAACGATACAGAGAAGATT
U6: R	GGAACGCTTCACGAATTTG

**Table 2:** Clinicopathological features and PSMA3-AS1 expression in OSCC patients.

Parameters	Group	Total	PSMA3-AS1 expression		*P* value
			High	Low	
Gender	Male	63	30	33	0.448
	Female	72	39	33	
Age (years)	<55	65	35	30	0.540
	≥55	70	34	36	
Tumor size	T1-T2	80	39	41	0.508
	T3-T4	55	30	25	
Distant metastasis	Yes	46	32	14	0.014
	No	79	37	42	
TNM stage	I-II	75	35	40	0.019
	III-IV	50	34	16	

**Table 3:** Multivariate Cox regression analysis for prognosis of OSCC patients.

Variable	Overall survival			Disease-free survival		
	HR	95% CI	*P* value	HR	95% CI	*P* value
Gender	1.322	0.562-1.776	0.323	1.213	0.654-1.891	0.291
Age	1.032	0.457-1.895	0.441	1.135	0.564-2.033	0.244
Tumor size	1.563	0.776-2.211	0.213	1.673	0.833-2.341	0.141
Distant metastasis	3.131	1.323-4.783	0.008	3.334	1.532-5.211	0.003
TNM stage	2.775	1.234-4.563	0.014	2.993	1.433-4.763	0.011
PSMA3-AS1 expression	2.987	1.341-4.453	0.016	3.034	1.563-4.873	0.006

## Data Availability

The datasets analyzed during the current study are available from the corresponding author on reasonable request.

## Abbreviations

OSCC: Oral squamous cell carcinoma
PSMA3-AS1: PSMA3 antisense RNA 1
ceRNA: Competitive RNA
NC: Negative control
CCK-8: Cell counting kit-8
EdU: 5-Ethynyl-2′-deoxyuridine
WT: Wild type
MUT: Mutant
OS: Overall survival
DFS: Disease-free survival
EMT: Epithelial-to-mesenchymal transition.
